# Improved overall survival in dendritic cell vaccination-induced immunoreactive subgroup of advanced melanoma patients

**DOI:** 10.1186/1479-5876-4-36

**Published:** 2006-08-16

**Authors:** Ruggero Ridolfi, Massimiliano Petrini, Laura Fiammenghi, Monica Stefanelli, Laura Ridolfi, Michela Ballardini, Giuseppe Migliori, Angela Riccobon

**Affiliations:** 1 Department of Medical Oncology, Morgagni-Pierantoni Hospital, Via Forlanini 34, 47100 Forlì, Italy; 2 Istituto Oncologico Romagnolo, Via Forlanini 34, 47100 Forlì, Italy; 3 Blood Transfusion Unit, Morgagni-Pierantoni Hospital, Via Forlanini 34, 47100 Forlì, Italy

## Abstract

**Background:**

We present our experience of therapeutic vaccination using dendritic cells (DC) pulsed with autologous tumor antigens in patients with advanced melanoma.

**Methods:**

Twenty-one pretreated advanced melanoma patients were vaccinated with autologous DC pulsed with 100 μg/ml of autologous-tumor-lysate (ATL) or – homogenate (ATH) and 50 μg/ml of keyhole limpet hemocyanin (KLH). The first 8 patients were treated subcutaneously or intradermally with immature-DC (iDC) (range 4.5 – 82 × 10^6^) and the remaining 13 intradermally with *in vitro *matured DC (mDC) (range 1.2–26 × 10^6^). Subcutaneous interleukin-2 (3 × 10^6 ^IU) was administered from days 3 to 7 of each treatment cycle.

**Results:**

Three of the 8 iDC patients obtained stabilizations (SD), each of 6 months' duration. The 13 mDC patients showed 1 complete response (8 months), 1 partial response (3 months), 2 mixed responses (6 and 12 months) and 3 SD (9, 7+, and 3+ months). Overall responses (OR) were observed in 4/21 (19%) patients, or 4/13 (30.7%) considering mDC treatment only. 10/21 (47.6%) patients showed non progressive disease (NPD), with 7/13 (53.8%) cases of NPD for mDC-treated patients. No major toxicities were observed. The positive delayed-type hypersensitivity (DTH) test to ATL/ATH and/or KLH correlated with increased overall survival (OS). Median OS was 24 months (range 3 – 45) for the 10 DTH-positive (1 iDC and 9 mDC) and 5 months (range 3–14) for the 11 DTH-negative patients (P < 0.001). The *in vitro *evaluation of gamma IFN-secreting T-cells in 10 patients showed good correlation with both DTH (75%) and clinical outcome (70%).

**Conclusion:**

Vaccination using DC pulsed with ATL/ATH and KLH in advanced melanoma patients is well tolerated and can induce a clinical response, especially when mDC are used. Successful immunization, verified by positive DTH, leads to longer survival.

## Background

In the complex interaction of relationships that exist between the immune system and cancer, therapeutic vaccination can be considered as a valid approach to overcoming the established state of immunotolerance between the two systems [1,2].

The use of dendritic cells (DC) derived from peripheral blood precursors and pulsed with tumor antigens forms the basis of experimental and clinical trials on antitumor vaccinations [3,4]. Although numerous clinical studies have reported interesting objective regressions, case series are often small with limited overall response rates [5]. A recent review on antitumor vaccines, especially with regard to melanoma, attributed an overall response (OR) of 3.8%, with an *encouraging *7.1% for DC vaccines [6].

Although OR rates for vaccination are somewhat limited, results obtained with DC vaccinations can be considered promising, also because of the low toxicity observed [7]. The majority of these studies have aimed at demonstrating immunization against a single or a cocktail of well defined antigens and, considering the potentially enormous number of antigens presented by the tumor and remodelled over time [8,9], it is rather surprising that any clinical responses have been observed at all [10-13]. However, in addition to the fundamental choice of antigens, the generation, cell culture, maturation status, and administration modalities of DC represent other critical steps needed for the good outcome of therapeutic vaccination.

At present, most authors use leukapheresis as source of DC [14-16]. Furthermore, the initial use of immature DC (iDC) has been replaced by that of DC matured *in vitro *(mDC) [17,18]. Conversely, the number of DC to administer, vaccination scheduling and duration, and routes of administration have still to be defined [19-21].

Another critical and much discussed issue is the clinical use of cytokines as vaccination adjuvants. In particular, it is not clear whether the administration of interkeukin-2 (IL-2) is necessary, useful, or counterproductive for clinical results [22-26].

It is also difficult to carry out a critical review of DC studies because the evaluation criteria used (WHO, RECIST) do not adapt well to some clinical situations obtained with vaccination [27,28]. The problem could, in part, be resolved by carrying out immunomonitoring, which is feasible for vaccinations with one or a small number of defined antigens, but much more complex when undefined antigen complexes are used [29].

We present an in-depth description of a phase I-II study of therapeutic vaccination using DC pulsed with autologous tumor lysate (ATL) or homogenate (ATH) and with keyhole limpet hemocyanin (KLH) in advanced melanoma patients. The study was begun in 2001 and was thus influenced by our own experience and by that of other authors.

## Methods

### Patients

From August 2001 to September 2005, 21 patients with advanced melanoma were entered onto the vaccination protocol using autologous DC pulsed with ATL or ATH and KLH. Eight patients were treated with immature DC (iDC) and 13 with mature DC (mDC). Inclusion criteria were age < 70 years, histologically confirmed diagnosis of melanoma, measurable disease (excluding brain metastases), previous removal of one or more metastatic lesions from which a sufficient quantity of ATL/ATH was obtained for at least 6 vaccinations, Performance Status (PS) ≤ 2 (according to ECOG criteria), life expectancy more than 4 months.

### Treatment

The first 8 patients were treated with iDC (range 4.5 – 82 × 10^6^) via subcutaneous or intradermal injection with an insulin needle. Several inoculations were made (average 6–8) near the groin or the armpit in non metastatic, non lymphadenectomized sites. A further 13 patients were treated with intradermal mDC (range 1.2–26 × 10^6^) alone following the abovementioned criteria, but with 8-10 inoculations. Subcutaneous IL-2 (Chiron, Milan, Italy) 3,000,000 IU/day was administered about 48 hours after each vaccination for 5 consecutive days. The first 4 vaccinations were carried out at intervals of 15 days and once a month thereafter.

Clinical evaluation (in partial acordance with RECIST criteria [28]), and delayed-type hypersensitivity (DTH) assessment were carried out before the first vaccination, after the 4th vaccination, and every 2 vaccinations thereafter. The disappearance of, or an important reduction in pre-existing lesions in concomitance with disease progression in other sites, was considered as a mixed response (MR). If progression did not compromise the patient's general conditions, several more vaccinations were carried out until progression was irrefutably documented. The best response obtained was considered for evaluation purposes. Toxicity or adverse events were assessed after each vaccine administration.

The study protocol was reviewed and approved by the local Ethics Committee, in accordance with ethical standards laid down in the 1964 Declaration of Helsinki, and authorized in July 2001 by the Italian Ministry of Health. All patients gave their informed written consent to receive treatment.

### Autologous Tumor Lysate (ATL) preparation

Surgically removed tumor samples were mechanically dispersed to create a single-cell suspension. The largest pieces were incubated at 37°C in enzyme mix (collagenase 0.1%, hyaluronidase 0.01%, DNAse 0.1%, Sigma, Milan, Italy) in RPMI 1640, (PAA Laboratories GmbH, Pasching, Austria) for 3 hours. At the end of incubation the pellets were washed 3 times with PBS and incubated for at least 20 minutes in sterile distilled water. Lysis was monitored by light microscope. Larger particles were removed by centrifugation (10 min at 600 *g*) and the supernatant was passed through a 0.2-μm filter. Protein contents were determined and aliquots were stored at -80°C until use, after verification of sterility.

### Autologous Tumor Homogenate (ATH) preparation

In some cases, the surgically removed tissue was stored at -80°C because the decision to vaccinate had still not been taken. Frozen tissue fragments were pulverized in a dismembrator after immersion in liquid nitrogen. Pulverized tissue was then suspended in PBS. After centrifugation, the supernatant was treated as described above.

### DC generation

DC were prepared from peripheral blood monocytes (PBMC) obtained by leukapheresis without previous mobilization. Five to nine liters of blood were processed in each collection. PBMC were purified on Ficoll-Paque. An aliquot of PBMC was utilized immediately for DC generation and the rest was frozen in bags for use at a later date (4–5 bags/each collection).

PBMC were incubated in tissue culture flasks with CellGro DC medium (Cell Genix, Freiburg, Germany) at 10 × 10^6 ^cells/ml for 2 h. The non-adherent cells were discarded and the adherent cells were incubated in CellGro DC medium containing 1000 IU/ml rhIL-4 (Cell Genix, Freiburg, Germany) and 1000 IU/ml rhGM-CSF (Schering-Plough, Milan, Italy) for 7 days to generate a DC-enriched cell population. On day 6, 90% of the DC culture was pulsed with ATL/ATH (100 mg/ml), while the remaining 10% was pulsed with KLH (50 mg/ml). Both cultures were then incubated overnight. On day 7, the cells were defined as immature DC (iDC). After eliminating the previous culture medium, pulsed iDC were cultured for a further 2 days with a cocktail of cytokines (TNFα, IL-1β, IL-6, Endogen, Pierce Biotechnology, Rockford, USA; PGE_2_, Cayman Chemical, Ann Arbor, MI, USA). On day 9 they were defined as mature DC (mDC). iDC or mDc were removed, washed and suspended in sterile saline for therapeutic infusion into the patient (iDC, range 4.5 – 82 × 10^6^; mDc, range 1.2–26 × 10^6^).

### Phenotype analysis

iDC and mDC phenotypes were determined by single or two-color fluorescence analysis in cells labelled with monoclonal antibodies (mAbs) and fixed in paraformaldehyde 2%. The fluorescence was analyzed by a FACS Vantage flow cytometer (Becton Dickinson, Milan, Italy). mAbs specific for human CD1a, CD14, CD80, CD86, HLA-DR (Becton Dickinson), CD83 (Immunotech, Marseille, France) and CCR7 (BD Pharmingen, Milan, Italy) were used.

### In Vivo monitoring

ATL or ATH (10 μg) and KLH (5 μg) were each suspended in 500 μl of PBS and injected intradermally into the forearm of the patient. PBS alone was used as negative control.

### In Vitro immunomonitoring

#### Evaluation of interferon-gamma-secreting cells

The patient's lymphocytes were collected and frozen before the first vaccination, after 4–6 vaccinations, and after 8–13 vaccinations. After thawing, 10 × 10^6 ^lymphocytes were stimulated *in vitro *overnight with ATL/ATH and 10 × 10^6 ^with KLH. Positive and negative controls were always included. Interferon-gamma (IFN-γ) secreting cells were detected using the IFN-γ Secretion Assay Cell Enrichment and Detection Kit (Miltenyi Biotec, Bergisch Gladbach, Germany) according to the manufacturer's instructions.

### Statistical evaluations

Survival time was calculated as the time between the date of the first cycle of therapy and the date of death from any cause. Survival curves were traced by the Kaplan-Meier method and the comparison between the two groups was based on the Log-Rank test. A two- sided P value of 5% was considered as significant. All analyses were performed using the R statistical software package.

## Results

Patient characteristics were as follows: 13 males, 8 females, with a median age of 52 years (range 35–75). Pretreatments, sites of evaluable disease, and PS are analytically described and analyzed in Table 1, together with the HLA determination of each patient. Two patients older than 70 years but in good general conditions were enrolled onto the study and treated on a compassionate basis.

**Table 1:** Patient characteristics

**Patient ID**	**Sex**	**Age (Years)**	**PS (ECOG)**	**HLA**	**Site of Evaluable Disease**	**Pretreatments**
1 L.A.	M	75	1	A_2_A_32_Cw_4_Cw_6_	spleen, soft tissue	IFN
2 G.I.	F	66	0	A_24_A_11_B_44_B_18_Bw_4_Bw_6_Cw_5_Cw_7_	pelvis	CT+RT
3 M.G.	M	73	1	A_1_A_10_B_13_B_41_Bw_4_Bw_6_	liver, lymph node	CT
4 S.P.	M	56	1	A_9_A_32_B_38_B_55_Bw_4_Bw_6_Cw_3_	lung, bone, lymph node	BIOCT
5 G.C.	M	51	0	A_1_A_2_B_8_B_35_Bw_6_Cw_4_Cw_7_	liver, lymph node	IFN
6 Z.V.	M	42	1	A_1_A_19_B_51_B_14_	lung	BIOCT
7 G.L.	M	56	2	A_3_A_28_B_35_B_53_Cw_4_	liver	BIOCT
8 B.A.	M	35	2	A_2_A_9_B_7_B_15_Bw_6_Cw_3_Cw_7_	lung, bone skin	BIOCT



9 P.M.	M	45	0	A_1_A_9_B_17_Bw_4_Bw_6_Cw_3_Cw_4_	lung, lymph node	BIO, BIOCT
10 P.M.	M	52	0	A_11_A_31_B_14_B_60_Bw_6_Cw_3_	kidney, adrenal gland	BIOCT
11 R.L.	F	46	1	A_3_A_29_B_44_Bw_4_	lung, liver, soft tissue	CT
12 G.D.	M	46	0	A_3_A_28_B_21_B_35_Cw_4_	lymph node	NT
13 R.G.	M	65	1	A_10_B_8_B_38_Bw_4_Bw_6_Cw_7_	lung, soft tissue	BIOCT
14 T.A.	F	61	1	A_2_A_19_B_35_B_37_Bw_4_Bw_6_Cw_4_	soft tissue	CT+RT
15 B.A.	F	59	2	A_2_A_9_B_39_B_44_Bw_4_Bw_6_Cw_5_	liver, soft tissue	BIOCT, locoregional CT
16 C.P.	F	39	0	A_9_A_19_B_14_B_44_Bw_4_Bw_6_Cw_5_	kidney, soft tissue	Locoregional CT
17 O.M.	M	56	1	A_19_A_28_B_5_B_16_Bw4Bw_6_	lung, soft tissue	BIOCT
18 L.B.	F	39	2		pelvis, lymph node	BIOCT
19 M.J.L.	F	37	0		lung, kidney, lymph node, soft tissue	BIOCT
20 O.G.	M	65	2	A_23_A_32_	adrenal gland, lymph node, soft tissue	BIOCT
21 M.R.	F	38	0		lymph node	BIOCT

					21 Viscera:	13 BIOCT
					- 1 spleen	3 BIO
					- 5 liver	4 CT
					- 8 lung	2 Locoregional CT
					- 3 kidney	2 RT
					- 2 adrenal gland	1 NT
					- 2 pelvis	
					2 Bone	
					8 Lymph node	
					9 Soft tissue	

PS (ECOG) = performance status according ECOG; CT= chemotherapy; RT= radiotherapy;

BIO=Immunotherapy (Interferon, Inteleukin-2); BIOCT= chemotherapy+immunotherapy; NT= no treatment.

Male/Female 13/8

Median age 52 years (35–75).

P.S.: 0 = 8 ; 1 = 8; 2 = 5

The number of cells administered for each vaccination was about 10 × 10^6^, with a higher median for iDC (17 × 10^6^, range: 11–49.5) than for mDC (9.5 × 10^6^, range: 5.9–12.6). A total of 145 administrations (49 iDC and 96 mDC) were made.

DC phenotype characteristics are shown in Table 2. iDC and mDC phenotyping was similar to that described in the literature, and there were no relevant differences in DC obtained from fresh or frozen tissue [4].

**Table 2:** Surface expression of DC markers in infused DC*

**Marker**	**iDC** Median % (range)	**mDC** Median % (range)
**CD1A**	**15.5 **(0 – 58)	**7 **(0 – 77)
**CD14**	**0 **(0 – 17)	**4 **(0 – 67)
**CD80**	**0 **(0 – 25)	**62 **(2 – 98)
**CD83**	**0 **(0 – 25)	**47 **(2 – 86)
**CD86**	**30 **(0 – 90)	**71 **(5 – 96)
**HLA-DR**	**51.5 **(3 – 95)	**78 **(13 – 100)
**CCR7**	**2.5 **(0 – 47)^§ ^	**45 **(1 – 91)

iDC, immature dendritic cells; mDC: mature dendritic cells.

^§^value calculated on 3/8 patients vaccinated with iDC.

*Data represent the percentage of positive cells out of the total number of DC analyzed.

DC were pulsed with ATH in 9 cases, with ATH + ATL in 2 patients, and with ATL in 10 patients. During the course of the study we decided to continue using, where possible, only ATL-pulsed DC (ATL should, in theory, have a higher percentage of antigenic protein).

### Clinical results

In the 8 patients treated with iDC, we observed 3 stabilizations (SD), each of 6 months' duration, and 5 PD. Overall survival (OS) of the SD patients was 45, 26 and 7 months, whereas that of the PD patients ranged from 4 to 10 months. DTH positivity showed a weak response for both ATH and KLH in the patient with the longest survival (median OS 7.5 months, range 4–45). A weak positivity to only KLH was seen in the patient with a 26-month survival, whereas no response was observed in the remaining patients (Table 3A).

**Table 3A:** Immunological and clinical response in patients vaccinated with iDC

**Patient ID**	**No. of Vaccinations**	**Antigens (Type of Lysate)**	**Median No. of Administered Cells × 10**^6 ^**(range)**	**DTH Best Response after 4 or More Vaccinations**.		**Vitiligo**	**Clinical Response**	**Response Duration (Months)**	**Overall Survival (Months)**
				L/H	KLH				
1 L.A.	8	H	49.5 (15 – 82)	+	+		**SD**	6	45
2 G.I.	5	L	13 (4.5 – 26)	-	+		**SD**	6	26
3 M.G.	5	L	11 (7 – 16.2)	-	-	+/-	**SD**	6	7
4 S.P.	8	L	26.5 (10 – 43.5)	-	-		PD	-	8
5 G.C.	6	L	19 (9.6 – 32)	-	-		PD	-	10
6 Z.V.	5	L/H	11.3 (6.4 – 21.5)	-	-		PD	-	4
7 G.L.	6	H	22.6 (16.4 – 48)	-	-		PD	-	5
8 B.A.	4	H	15 (12 – 51)	-	-		PD	-	5

**No. of Patients**	**Administration Route**	**Site**	**No. of Inoculations**						
						
8 iDC	4 i.d. and 4 s.c.	6 inguinal and 2 axillary	6–8						

DTH, delayed-type hypersensitivity; L, autologus lysate; H, autologous homogenate; KLH, keyhole limpet hemocyanin; SD, stabilization; PD, progressive disease.

i.d., intradermal; s.c., subcutaneous

In the 13 mDC-treated patients, one complete response (CR) of 8 months' duration (OS = 32+ months), one partial response (PR) lasting 3 months (OS = 14 months) and 2 mixed responses (MR) of 12 and 6 months' duration (OS = 28+ and 22 months, respectively) were obtained. There were also 3 SD lasting 7+, 6 and 3+ months (the third patient is still undergoing treatment) (OS = 7+, 14 and 3+ months, respectively) and 6 PD with a median OS of 5.5 months (range 3–20). Median OS was 8 months (range 3–32+).

**Figure 1:**
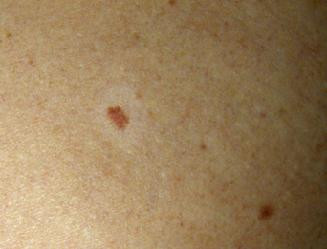
Onset of vitiligo in patient no.12 who had CR after treatment with mDC.

**Figure 2:**
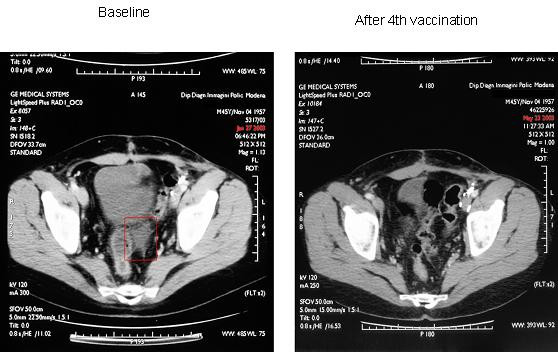
Baseline and post 4^th ^vaccination CT scan of patient no. 12, treated with mDC. The patient had an 8-month CR of the abdominal soft tissue lesions.

Patient no. 12 (46-year old man), who had numerous metastatic abdominal and pelvic lymph node localizations, obtained a CR of 8 months, and was the only patient who had not received previous treatment. He developed vitiligo (Figure 1) and the DTH test confirmed a good response to ATL and KLH. Baseline and first-evaluation CT scan images are shown in Figure 2. Patient no. 9 rapidly obtained a PR for lung and lymph node lesions but developed brain metastases after only 3 months. MR was observed in patient nos. 10 and 14, in whom subcutaneous and lymph node metastases disappeared and reappeared for 6 and 12 months, respectively. These patients showed positive DTH for KLH, the former also a strong positive reaction for ATL. All data are analytically reported in Table 3B.

**Table 3B:** Immunological and clinical response in patients vaccinated with mDC

**Patient ID**	**No. of Vaccinations**	**Antigens (Type of Lysate)**	**Median No. of Administered Cells × 10**^6 ^**(range)**	**DTH Best Response after 4 or More Vaccinations**		**Vitiligo**	**Clinical Response**	**Response Duration (Months)**	**Overall Survival (Months)**
				L/H	KLH				
9 P.M.	7	L	11.4 (9 – 24.1)	-	-		**PR**	3	14
10 P.M.	15	L	15 (2.8 – 24)	++	++++		**MR**	6	22
11 R.L.	10	H	9.5 (4 – 13.4)	-	++		**SD**	9	14
12 G.D.	16	L/H	12.6 (2.8 – 20.8)	++	+++	+	**CR**	8	32+
13 R.G.	4	H	9.3 (8 – 26)	-	+++		PD	-	8
14 T.A.	13	H	9 (1.2 – 12)	-	++		**MR**	12	28+
15 B.A.	4	H	5.9 (3.7 – 12)	-	-		PD	-	7
16 C.P.	6	H	7.8 (1.6 – 15)	-	++		PD	-	20
17 O.M.	4	H	11.5 (10 – 21)	-	-		PD	-	5
18 L.B.	4	L	12.5 (10 – 15.5)	-	-		PD	-	3
19 M.J.L.	8	L	6 (2.2 – 10)	+	+++	+	**SD**	7+	7+
20 O.G.	5	L	10 (8.8 – 12.3)	-	-		PD	-	3
21 M.R.	4	L	9.2 (8 – 10)	+	++		**SD (u.t.)**	3+	3+

**No. of Patients**	**Administration Route**	**Site**	**No. of Inoculations**						
						
13 mDC	13 i.d.	10 inguinal and 3 axillary	8–10						

DTH, delayed-type hypersensitivity; L, autologous lysate; H, autologous homogenate; KLH, keyhole limpet hemocyanin.

PD, progressive disease; MR, mixed response; SD, stabilization; PR, partial response; CR, complete response; u.t., undergoing treatment.

i.d., intradermal; s.c., subcutaneous

The OR percentage for the 21 patients treated was 19%, which increased to 30.7% (4/13) when only the results obtained with mDC were considered. The overall percentage of patients who did not progress was 47.6% (10/21), rising to 53.8 % (7/13) for those treated with mDC. Our data do not seem to have been influenced by age, sex, or HLA distribution (in 8/10 patients there was a distribution of 2 patients for each of the principle alleles, A1, A2, A3 and A11). There does not appear to be any specific correlation between response and site of disease or type of pretreatment, although it should be remembered that the only patient who obtained a CR had not been pretreated. Futhermore, the number of cells injected would not appear to affect response.

Conversely, initial PS may have exerted an influence on response; all 5 patients with PS = 2 at baseline progressed. Clinical response obviously correlated positively with OS: 11 patients with progressive disease (PD) had a median OS of 5 months (range 3–20), whereas the 10 who did not progress had a median OS of 21 months (range 3^+ ^– 45).

### Toxicity

Apart from swelling, redness and pruritus around the site of inoculation, no noteworthy toxicities or side-effects were observed. A low fever with mild flu-like symptoms (grade 1–2) accompanied the administration of IL-2 from the 3rd to the 7th day of treatment. No autoimmune phenomena were observed apart from the onset of vitiligo in 2 patients and the flaring up of a pre-existing vitiligo in a third patient.

### In Vivo immunomonitoring

**Figure 3:**
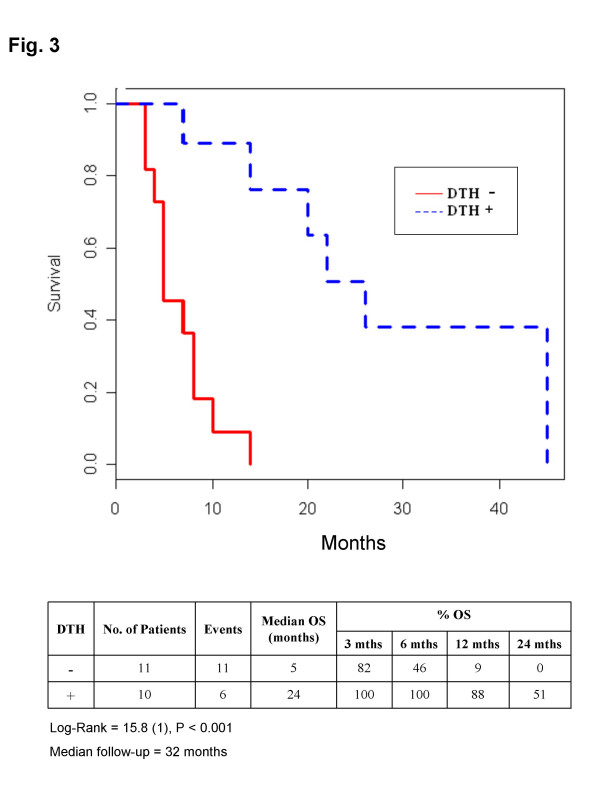
Actuarial curves of overall survival (OS) based on DTH results. Eleven patients had negative DTH with a median OS of 5 months and no survival at 24 months. Ten patients had positive DTH (for ATL/ATH and/or for KLH) and a median OS of 24 months, with 50% still alive after 2 years (P < 0.001).

Amongst the 10 patients with non progressive disease (NPD), only 2 had completely negative DTH reactions for both KLH and ATL/ATH, whereas the 5 patients who developed a response to ATL/ATH were all responders. Correlating this with OS, it can be seen that, amongst patients with a positive DTH reaction (10 patients, 8 of whom had NPD), median survival was 24 months (range 3^+^-45), whereas those with negative DTH (11 patients, 2 of whom had NPD), had a median OS of 5 months (range 3–14). The actuarial curve of survival in relation to a positive or negative DTH reaction shows that 24-month OS was 51% in DTH+ patients compared to 0% in DTH- patients (P < 0.001) at a median follow-up of 32 months (Figure 3). None of the patients who began treatment with a PS of 2 showed a positive DTH test.

### In Vitro immunomonitoring

*In vitro *immunomonitoring was performed by evaluating the number of lymphocytes in peripheral blood that are capable of producing gamma-IFN after stimulation with KLH and ATL/ATH. The test was only commercialized recently and was carried out on 10 patients (1 treated with iDC and 9 with mDC).

Clinical responses in 7 patients corresponded with the indications of the test (70%): positivity was correlated with NPD in 4 cases and with PD in 3 patients. Comparing the results from the *in vitro *test with those of DTH stimulation using both KLH and ATL/ATH, overall concordance was 75% (Table 4).

**Table 4:** *In vitro *immunomonitoring (Miltenyi Test): comparison between IFN-γ secreting lymphocytes after stimulation with KLH and ATL/ATH, and DTH test and clinical outcome

**Patient ID**	**Stimulation**	**Baseline**	**After 4/6 Vaccinations**	**After 8/13 Vaccinations**	**DTH**	**Clinical Response**
4 S.P.^a^	KLH	0	0		**Negative**	**PD**
	L	0	0		**Negative**	
9 P.M.^b^	KLH		0		**Negative**	PR
	L		0		**Negative**	
10 P.M.^b^		KLH	0	0	186	**Positive**	**MR**
	L	0	0	0	Positive	
12 G.D.^b^		KLH	133	1238	2253	**Positive**	**CR**
	L	56	456	13405	**Positive**	
14 T.A.^b^	KLH	4	356	73	**Positive**	**MR**
	H	0	0	0	Negative	
16 C.P.^b^	KLH	0	103		Positive	PD
	H	0	0		**Negative**	
18 L.B.^b^	KLH	264	110		**Negative**	**PD**
	L	0	0		**Negative**	
19 M.J.L.^b^		KLH	0	0		Positive	SD
	L	0	0		Positive	
20 O.M.^b^		KLH	0	0		**Negative**	**PD**
	L	0	0		**Negative**	
21 M.R.^b^		KLH	0	99		**Positive**	**SD**
	L	0	292		**Positive**	

DTH, delayed-type hypersensitivity; L, autologous tumor lysate; H, autologous tumor homogenate; KLH, keyhole limpet hemocyanin.

^a ^= pts treated with iDC.

^b ^= pts treated with mDC.

**bold **= data corresponding to test

## Discussion

The practice of antitumor vaccination with DC has brought with it new hope, especially for patients with melanoma, who obtain unsatisfactory results from traditional therapies [30,31]. The high number of known tumor antigens makes melanoma a good target for vaccine therapy [8]. DC vaccination is generally easy to administer and induces low toxicity, but studies published to date are difficult to compare with each other because of the small cases series involved, the limited number of clinical responses observed, and the numerous methodologies of DC preparation used: Cranmer, in fact, recently listed up to 12 possible variations in culture methodology and treatment modalities [32].

Crucial issues such as the best antigens or the most effective evaluation criteria to use have still to be clarified. DC derived from peripheral blood rather than from bone marrow tend to be used and it has been seen that mDC are undoubtedly better at migrating to regional lymph nodes and are more effective at determining an immunological and clinical response than iDC [14,33,34]. It has also recently been reported that intradermal administration results in a higher DC migration than subcutaneous injection [21,35].

However, one of the main problems of vaccination therapy remains the type of antigens to use. Pulsing DC with known single peptides enables us to accurately monitor immunization, but literature results highlight poor, short-term clinical responses, probably due to tumor escape [36,37]. Whilst pulsing with tumor extracts (lysate, apoptotic bodies, heat shock proteins, etc.) theoretically implies that all tumor antigens are represented, it also complicates the process of specific immunomonitoring, requires sizeable quantities of tumor tissue, and results in DC being loaded with a large number of other non antigenic proteins that could reduce, in terms of percentages, the efficacy of the exposure [6,38-41]. Novel methods of pulsing with tumor RNA or with a selection of RNA antigens could perhaps result in increased immune efficacy, especially if we bear in mind the large number of melanoma-restricted genes that have already been identified and codified [9,42].

Clinical evaluation also poses a problem. There is a growing need to modify RECIST criteria, widely used in oncology, when assessing clinical responses in vaccine therapy, *e.g. *to define *mixed *responses or responses that occur after an initial progression. Once a vaccine strategy has been clarified or better defined, a modified integrated system of clinical evaluation will be needed [43].

New strategies must also be sought to overcome tumor immunosuppression [44,45]. The use of lymphoablative chemotherapy has been hypothesized to reduce or eliminate the component of T-regulator lymphocytes before vaccination [46-49]. Studies in this area could also clarify the still controversial role of IL-2 as a vaccine adjuvant [50-53] and could help to provide valuable information about new molecular targets or cytokines [54,55].

Recent publications would seem to indicate that the presence and/or activation of specific lymphocytes induced by immunostimulation correlates positively with survival [56-59]. Our results concur with this finding and also indicate the need for a better selection of patients, especially in view of the fact that poor PS would seem to correlate with no clinical or immunological response. Patients with minimal residual disease or evidence of probable immunocompetence can be considered candidates for controlled vaccine trials [60,61].

In conclusion, although vaccination treatment with DC pulsed with KLH and ATL/ATH is an experimental approach and still requires numerous adjustments and verifications, it would seem to be practicable, non toxic, and most importantly, effective in prolonging OS if administered in a subgroup of patients who show immunoreactivity.

## Abbreviations

DC, dendritic cell; OR, overall response; iDC, immature dendritic cell; mDC, mature dendritic cell; ATL, autologous tumor lysate; ATH, autologous tumor homogenate; KLH, keyhole limpet hemocyanin; DTH, delayed-type hypersensitivity; PS, Performance Status; MP, mixed response; PBMC, peripheral blood monocytes; IFN-γ, interferon-gamma; SD, stabilization; NPD, non-progressive disease.

## Competing interests

The author(s) declare that they have no competing interests.

## Authors' contributions

RR and LR participated in the design of the study and were responsible for the clinical side of the study. AR, MP, LF and MS also participated in the study design and were responsible for the biological part of the study. GM performed the apheresis collections. MB performed the mathematical and statistical analysis. All authors read and approved the final manuscript.

## References

[B1] Banchereau J, Palucka AK, Dhodapkar M, Burkeholder S, Taquet N, Rolland A, Taquet S, Coquery S, Wittkowski KM, Bhardwaj N, Pineiro L, Steinman R, Fay J (2001). Immune and clinical responses in patients with metastatic melanoma to CD34(+) progenitor-derived dendritic cell vaccine. Cancer Res.

[B2] Ichim CV (2005). Revisiting immunosurveillance and immunostimulation: Implications for cancer immunotherapy. J Transl Med.

[B3] O'Neill DW, Adams S, Bhardwaj N (2004). Manipulating dendritic cell biology for the active immunotherapy of cancer. Blood.

[B4] Banchereau J, Palucka AK (2005). Dendritic cells as therapeutic vaccines against cancer. Nat Rev Immunol.

[B5] Ridgway D (2003). The first 1000 dendritic cell vaccinees. Cancer Invest.

[B6] Rosenberg SA, Yang JC, Restifo NP (2004). Cancer immunotherapy: moving beyond current vaccines. Nat Med.

[B7] Kaufman HL (2005). Integrating bench with bedside: the role of vaccine therapy in the treatment of solid tumors. J Clin Oncol.

[B8] Novellino L, Castelli C, Parmiani G (2005). A listing of human tumor antigens recognized by T cells: March 2004 update. Cancer Immunol Immunother.

[B9] Wang E, Panelli MC, Zavaglia K, Mandruzzato S, Hu N, Taylor PR, Seliger B, Zanovello P, Freedman RS, Marincola FM (2004). Melanoma-restricted genes. J Transl Med.

[B10] Mackensen A, Herbst B, Chen JL, Kohler G, Noppen C, Herr W, Spagnoli GC, Cerundolo V, Lindemann A (2000). Phase I study in melanoma patients of a vaccine with peptide-pulsed dendritic cells generated in vitro from CD34(+) hematopoietic progenitor cells. Int J Cancer.

[B11] Thurner B, Haendle I, Roder C, Dieckmann D, Keikavoussi P, Jonuleit H, Bender A, Maczek C, Schreiner D, von den Driesch P, Brocker EB, Steinman RM, Enk A, Kampgen E, Schuler G (1999). Vaccination with mage-3A1 peptide-pulsed mature, monocyte-derived dendritic cells expands specific cytotoxic T cells and induces regression of some metastases in advanced stage IV melanoma. J Exp Med.

[B12] Schuler-Thurner B, Dieckmann D, Keikavoussi P, Bender A, Maczek C, Jonuleit H, Roder C, Haendle I, Leisgang W, Dunbar R, Cerundolo V, von Den Driesch P, Knop J, Brocker EB, Enk A, Kampgen E, Schuler G (2000). Mage-3 and influenza-matrix peptide-specific cytotoxic T cells are inducible in terminal stage HLA-A2.1+ melanoma patients by mature monocyte-derived dendritic cells. J Immunol.

[B13] Pardoll D, Allison J (2004). Cancer immunotherapy: breaking the barriers to harvest the crop. Nat Med.

[B14] Banchereau J, Steinman RM (1998). Dendritic cells and the control of immunity. Nature.

[B15] Schuler G, Schuler-Thurner B, Steinman RM (2003). The use of dendritic cells in cancer immunotherapy. Curr Opin Immunol.

[B16] Ridolfi R, Ridolfi L, Petrini M, Fiammenghi L, Riccobon A (2003). Dendritic cell vaccination and immunostimulation in advanced melanoma. Expert Rev Vaccines.

[B17] Adams S, O'Neill D, Bhardwaj N (2004). Maturation matters: importance of maturation for antitumor immunity of dendritic cell vaccines. J Clin Oncol.

[B18] Sussman JJ, Parihar R, Winstead K, Finkelman FD (2004). Prolonged culture of vaccine-primed lymphocytes results in decreased antitumor killing and change in cytokine secretion. Cancer Res.

[B19] Panelli MC, Wunderlich J, Jeffries J, Wang E, Mixon A, Rosenberg SA, Marincola FM (2000). Phase 1 study in patients with metastatic melanoma of immunization with dendritic cells presenting epitopes derived from the melanoma-associated antigens MART-1 and gp100. J Immunother.

[B20] Lau R, Wang F, Jeffery G, Marty V, Kuniyoshi J, Bade E, Ryback ME, Weber J (2001). Phase I trial of intravenous peptide-pulsed dendritic cells in patients with metastatic melanoma. J Immunother.

[B21] Ridolfi R, Riccobon A, Galassi R, Giorgetti G, Petrini M, Fiammenghi L, Stefanelli M, Ridolfi L, Moretti A, Migliori G, Fiorentini G (2004). Evaluation of in vivo labelled dendritic cell migration in cancer patients. J Transl Med.

[B22] Shimizu K, Fields RC, Redman BG, Giedlin M, Mule JJ (2000). Potentiation of immunologic responsiveness to dendritic cell-based tumor vaccines by recombinant interleukin-2. Cancer J Sci Am.

[B23] Slingluff CL, Petroni GR, Yamshchikov GV, Hibbitts S, Grosh WW, Chianese-Bullock KA, Bissonette EA, Barnd DL, Deacon DH, Patterson JW, Parekh J, Neese PY, Woodson EM, Wiernasz CJ, Merrill P (2004). Immunologic and clinical outcomes of vaccination with a multiepitope melanoma peptide vaccine plus low-dose interleukin-2 administered either concurrently or on a delayed schedule. J Clin Oncol.

[B24] Zhang H, Chua KS, Guimond M, Kapoor V, Brown MV, Fleisher TA, Long LM, Bernstein D, Hill BJ, Douek DC, Berzofsky JA, Carter CS, Read EJ, Helman LJ, Mackall CL (2005). Lymphopenia and interleukin-2 therapy alter homeostasis of CD4+CD25+ regulatory T cells. Nat Med.

[B25] Andersen MH, Gehl J, Reker S, Geertsen P, Becker JC, thor Stratem P (2005). Concomitant administration of interleukin-2 during therapeutic vaccinations against cancer: the good, the bad, or the evil?. J Clin Oncol.

[B26] de la Rosa M, Rutz S, Dorninger H, Scheffold A (2004). Interleukin-2 is essential for CD4+CD25+ regulatory T cell function. Eur J Immunol.

[B27] Miller AB, Hoogstraten B, Staquet M, Winkler A (1981). Reporting results of cancer treatment. Cancer.

[B28] Therasse P, Arbuck SG, Eisenhauer EA, Wanders J, Kaplan RS, Rubinstein L, Verweij J, Van Glabbeke M, van Oosterom AT, Christian MC, Gwyther SG (2000). New guidelines to evaluate the response to treatment in solid tumors. European Organization for Research and Treatment of Cancer, National Cancer Institute of the United States, National Cancer Institute of Canada. J Natl Cancer Inst.

[B29] Keilholz U, Weber J, Finke JH, Gabrilovich DI, Kast WM, Disis ML, Kirkwood JM, Scheibenbogen C, Schlom J, Maino VC, Lyerly HK, Lee PP, Storkus W, Marincola F, Worobec A, Atkins MB (2002). Immunologic monitoring of cancer vaccine therapy: results of a workshop sponsored by the Society for Biological Therapy. J Immunother.

[B30] Atkins MB (1997). The treatment of metastatic melanoma with chemotherapy and biologics. Curr Opin Oncol.

[B31] Tsao H, Atkins MB, Sober AJ (2004). Management of cutaneous melanoma. New Engl J Med.

[B32] Cranmer LD, Trevor KT, Hersh EM (2004). Clinical applications of dendritic cell vaccination in the treatment of cancer. Cancer Immunol Immunother.

[B33] Nestle FO, Alijagic S, Gilliet M, Sun Y, Grabbe S, Dummer R, Burg G, Schadendorf D (1998). Vaccination of melanoma patients with peptide- or tumor lysate-pulsed dendritic cells. Nat Med.

[B34] Schuler-Thurner B, Schultz ES, Berger TG, Weinlich G, Ebner S, Woerl P, Bender A, Feuerstein B, Fritsch PO, Romani N, Schuler G (2002). Rapid induction of tumor-specific type 1 T helper cells in metastatic melanoma patients by vaccination with mature, cryopreserved, peptide-loaded monocyte-derived dendritic cells. J Exp Med.

[B35] Smithers M, O'Connell K, MacFadyen S, Chambers M, Greenwood K, Boyce A, Abdul-Jabbar I, Barker K, Grimmett K, Walpole E, Thomas R (2003). Clinical response after intradermal immature dendritic cell vaccination in metastatic melanoma is associated with immune response to particulate antigen. Cancer Immunol Immunother.

[B36] Andersen MH, Keikavoussi P, Brocker EB, Schuler-Thurner B, Jonassen M, Sondergaard I, Straten PT, Becker JC, Kampgen E (2001). Induction of systemic CTL responses in melanoma patients by dendritic cell vaccination: cessation of CTL responses is associated with disease progression. Int J Cancer.

[B37] Toungouz M, Libin M, Bulte F, Faid L, Lehmann F, Duriau D, Laporte M, Gangji D, Bruyns C, Lambermont M, Goldman M, Velu T (2001). Transient expansion of peptide-specific lymphocytes producing IFN-gamma after vaccination with dendritic cells pulsed with MAGE peptides in patients with mage-A1/A3-positive tumors. J Leukoc Biol.

[B38] Rosenberg SA, Yang JC, Schwartzentruber DJ, Hwu P, Marincola FM, Topalian SL, Restifo NP, Sznol M, Schwarz SL, Spiess PJ, Wunderlich JR, Seipp CA, Einhorn JH, Rogers-Freezer L, White DE (1999). Impact of cytokine administration on the generation of antitumor reactivity in patients with metastatic melanoma receiving a peptide vaccine. J Immunol.

[B39] Krause SW, Neumann C, Soruri A, Mayer S, Peters JH, Andreesen R (2002). The treatment of patients with disseminated malignant melanoma by vaccination with autologous cell hybrids of tumor cells and dendritic cells. J Immunother.

[B40] Chang AE, Redman BG, Whitfield JR, Nickoloff BJ, Braun TM, Lee PP, Geiger JD, Mule JJ (2002). A phase I trial of tumor lysate-pulsed dendritic cells in the treatment of advanced cancer. Clin Cancer Res.

[B41] O'Rourke MG, Johnson M, Lanagan C, See J, Yang J, Bell JR, Slater GJ, Kerr BM, Crowe B, Purdie DM, Elliott SL, Ellem KA, Schmidt CW (2003). Durable complete clinical responses in a phase I/II trial using an autologous melanoma cell/dendritic cell vaccine. Cancer Immunol Immunother.

[B42] Panelli MC, Martin B, Nagorsen D, Wang E, Smith K, Monsurro V, Marincola FM (2004). A genomic- and proteomic-based hypothesis on the eclectic effects of systemic interleukin-2 administration in the context of melanoma-specific immunization. Cells Tissues Organs.

[B43] Simon RM, Steinberg SM, Hamilton M, Hildesheim A, Khleif S, Kwak LW, Mackall CL, Schlom J, Topalian SL, Berzofsky JA (2001). Clinical trial designs for the early clinical development of therapeutic cancer vaccines. J Clin Oncol.

[B44] Marincola FM, Jaffee EM, Hicklin DJ, Ferrone S (2000). Escape of human solid tumors from T-cell recognition: molecular mechanisms and functional significance. Adv Immunol.

[B45] Taylor DD, Gercel-Taylor C, Lyons KS, Stanson J, Whiteside TL (2003). T-cell apoptosis and suppression of T-cell receptor/CD3-zeta by Fas ligand-containing membrane vesicles shed from ovarian tumors. Clin Cancer Res.

[B46] Dudley ME, Wunderlich JR, Robbins PF, Yang JC, Hwu P, Schwartzentruber DJ, Topalian SL, Sherry R, Restifo NP, Hubicki AM, Robinson MR, Raffeld M, Duray P, Seipp CA, Rogers-Freezer L, Morton KE, Mavroukakis SA, White DE, Rosenberg SA (2002). Cancer regression and autoimmunity in patients after clonal repopulation with antitumor lymphocytes. Science.

[B47] Dudley ME, Wunderlich JR, Yang JC, Sherry RM, Topalian SL, Restifo NP, Royal RE, Kammula U, White DE, Mavroukakis SA, Rogers LJ, Gracia GJ, Jones SA, Mangiameli DP, Pelletier MM, Gea-Banacloche J, Robinson MR, Berman DM, Filie AC, Abati A, Rosenberg SA (2005). Adoptive cell transfer therapy following non-myeloablative but lymphodepleting chemotherapy for the treatment of patients with refractory metastatic melanoma. J Clin Oncol.

[B48] Ahmadzadeh M, Rosenberg SA (2006). IL-2 administration increases CD4+CD25hiFoxp3+ regulatory T cells in cancer patients. Blood.

[B49] Powell DJ, Parker LL, Rosenberg SA (2005). Large-scale depletion of CD25+ regulatory T cells from patient leukapheresis samples. J Immunother.

[B50] Slingluff CL, Speiser DE (2005). Progress and controversies in developing cancer vaccines. J Transl Med.

[B51] Fontenot JD, Rasmussen JP, Gavin MA, Rudensky AY (2005). A function for interleukin 2 in Foxp3-expressing regulatory T cells. Nat Immunol.

[B52] Antony PA, Restifo NP (2005). CD4+CD25+ T regulatory cells, immunotherapy of cancer, and interleukin-2. J Immunother.

[B53] Malek TR, Bayer AL (2004). Tolerance, not immunity, crucially depends on IL-2. Nat Rev Immunol.

[B54] Dranoff G (2005). CTLA-4 blockade: unveiling immune regulation. J Clin Oncol.

[B55] Melchionda F, Fry TJ, Milliron MJ, McKirdy MA, Tagaya Y, Mackall CL (2005). Adjuvant IL-7 or IL-15 overcomes immunodominance and improves survival of the CD8+ memory cell pool. J Clin Invest.

[B56] Prasad SJ, Farrand KJ, Matthews SA, Chang JH, McHugh RS, Ronchese F (2005). Dendritic cells loaded with stressed tumor cells elicit long-lasting protective tumor immunity in mice depleted of CD4+CD25+ regulatory T cells. J Immunol.

[B57] Robbins PF, Dudley ME, Wunderlich J, El-Gamil M, Li YF, Zhou J, Huang J, Powell DJ, Rosenberg SA (2004). Cutting edge: persistence of transferred lymphocyte clonotypes correlates with cancer regression in patients receiving cell transfer therapy. J Immunol.

[B58] de Vries IJ, Bernsen MR, Lesterhuis WJ, Scharenborg NM, Strijk SP, Gerritsen MJ, Ruiter DJ, Figdor CG, Punt CJ, Adema GJ (2005). Immunomonitoring tumor-specific T cells in delayed-type hypersensitivity skin biopsies after dendritic cell vaccination correlates with clinical outcome. J Clin Oncol.

[B59] Escobar A, Lopez M, Serrano A, Ramirez M, Perez C, Aguirre A, Gonzalez R, Alfaro J, Larrondo M, Fodor M, Ferrada C, Salazar-Onfray F (2005). Dendritic cell immunizations alone or combined with low doses of interleukin-2 induce specific immune responses in melanoma patients. Clin Exp Immunol.

[B60] Stift A, Friedl J, Dubsky P, Bachleitner-Hofmann T, Schueller G, Zontsich T, Benkoe T, Radelbauer K, Brostjan C, Jakesz R, Gnant M (2003). Dendritic cell-based vaccination in solid cancer. J Clin Oncol.

[B61] Berd D, Sato T, Maguire HC, Kairys J, Mastrangelo MJ (2004). Immunopharmacologic analysis of an autologous, hapten-modified human melanoma vaccine. J Clin Oncol.

